# Psychometric Properties and Measurement Invariance of the Chinese Version of the Brief Assessment of Impaired Cognition Questionnaire in Community-Dwelling Older Adults

**DOI:** 10.3389/fpubh.2022.908827

**Published:** 2022-06-17

**Authors:** Shaojie Li, Guanghui Cui, Kasper Jørgensen, Zimi Cheng, Zihao Li, Huilan Xu

**Affiliations:** ^1^Xiangya School of Public Health, Central South University, Changsha, China; ^2^Department of Integrated Traditional Chinese and Western Medicine, Peking University First Hospital, Beijing, China; ^3^Danish Dementia Research Centre, Department of Neurology, University of Copenhagen, Copenhagen, Denmark; ^4^West China School of Public Health and West China Fourth Hospital, Sichuan University, Chengdu, China

**Keywords:** cognitive function, validation, reliability, measurement invariance, older adults, China

## Abstract

This study aimed to verify the Chinese version of the Brief Assessment of Impaired Cognition Questionnaire (C-BASIC-Q), and provide a new tool for the future large-scale epidemiological investigation of cognitive function in China. From March to May 2021, a cross-sectional study of 2,144 Chinese community-dwelling older adults (men = 1,075, mean age = 72.01 years, *SD* = 6.96 years, ranging from 60–99 years) was conducted in Jinan. Exploratory and confirmatory factor analyses were performed to evaluate the factor structure of the C-BASIC-Q. Convergent validity was evaluated by correlations with the Mini-Mental State Examination (MMSE). Internal consistency and test-retest reliability were evaluated using Cronbach's alpha and retest correlations in a sub-sample (*n* = 129). Linear regression was used to analyze the impact of demographic factors on the MMSE and C-BASIC-Q scores. Measurement invariance was evaluated using a multi-group confirmatory factor analysis. The mean C-BASIC-Q score was 15.94 (*SD* = 3.43). Factor analysis suggested a three-factor structure of C-BASIC-Q (self-report, orientation, and informant report). The C-BASIC-Q score was significantly positively associated with the MMSE score, showing good convergent validity. Cronbach's alpha of the C-BASIC-Q was 0.862, and the test-retest correlation coefficient was significant (*r* = 0.952, *p* < 0.001), indicating good internal consistency and test-retest reliability. Measurement invariance analysis showed that C-BASIC-Q had configural, metric, and scalar invariance across sex, age, residence, education level and marital status. C-BASIC-Q was less affected by age, residence, education, and marital status than the MMSE. In summary, the C-BASIC-Q had good reliability, validity, and measurement invariance, and is a valid tool for evaluating cognitive functioning in Chinese community-dwelling older adults.

## Introduction

As a result of economic development and the advancement of medical technology, the average life expectancy of human beings has gradually increased. Concomitantly, the degree of aging of the world's population has continued to increase (1), and age-related decline in cognitive functioning has become a public health problem worldwide. Mild cognitive impairment (MCI) is widely regarded by researchers as an intermediate phase between normal cognitive aging and overt dementia. Data from earlier epidemiological surveys showed that the prevalence of cognitive impairment without dementia was between 5.1 and 35.9% (2). A recent meta-analysis found that the incidence of MCI per 1,000 person-years was 22.5 for ages 75–79 years, 40.9 for ages 80–84 years, and 60.1 for ages 85+ years (3). Cognitive impairment has a great negative impact on the physical and mental health of older adults and their caregivers, not only because it can reduce their quality of life (4, 5), but it also creates a great care and economic burden (6, 7). Studies have found that cognitive impairment in the elderly was greatly affected by midlife or early age cognitive functioning (8). Therefore, early investigation of cognitive function has important public health implications for the prevention of cognitive impairment. In this field, the development and use of cognitive function assessment tools is a core issue.

Currently, cognitive function is generally evaluated by means of commonly used scales such as the Mini-Mental State Examination (MMSE), the Montreal Cognitive Assessment (MoCA), and the clock drawing test (CDT) (9, 10). Among them, the MMSE and MoCA are the most commonly used tools in this field, especially in China. A meta-analysis showed that the MMSE's pooled sensitivity was 0.89 (95% CI, 0.85 to 0.92) and specificity was 0.89 (95% CI, 0.85 to 0.93), which suggests good accuracy for detecting dementia (11). However, the MMSE is susceptible to the education level of participants, is prone to false-positive or false-negative results, and has poor sensitivity in identifying MCI and may not be suitable for screening for MCI in primary care and community research (12). In addition, a recent study found MMSE was less accurate in distinguishing MCI from subjective cognitive decline (SCD); meanwhile, even when the MMSE was used in combination with a quick test of cognitive speed, the MCI and SCD cannot be distinguished with sufficient accuracy (13). To mitigate these limitations of the MMSE, Nasreddine et al. compiled the MoCA specifically for MCI screening (14). A systematic review indicated that the MoCA was superior to the MMSE in identifying MCI (15). Meanwhile, previous study also found that MoCA was more efficacious in identifying subtle cognitive decline than MMSE (16). However, it should be noted that the difficulty level of the MoCA's items is higher, making it difficult for older participants with lower education levels to understand (17), resulting in lower scores which do not accurately reflect their level of cognitive functioning (18). In light of the differences in regional dialects and culture in China, the MoCA is available in multiple versions (19–23) and there is no uniformly recognized version and cut-off value (24–26). In addition, the MMSE and the MoCA have several common limitations, such as too many items, long measurement time, and a heavier survey burden on investigators and participants in community screening and large-scale epidemiological surveys. Therefore, it is necessary to introduce or develop a shorter Chinese version of a cognitive function assessment tool.

In 2019, Jørgensen et al. (27) combined cognitive testing with both patient and informant reports to develop a Brief Assessment of Cognitive Impairment (BASIC), a new brief case-finding tool for dementia and cognitive impairment. BASIC was a case-finding instrument in clinical settings, including patient-directed questions (three questions), Supermarket Fluency (one question), Category Cued Memory Test (one questions), and informant-directed questions (three questions), a total of eight questions (27). However, it should be noted that BASIC may not be appropriate in a community setting because its two cognitive tests take more time and was not easy to manage (need additional tools to cooperate with the test). For example, a stimulus card is needed for the Category Cued Memory Test, which may increase the investigation time and the burden of investigators (28). Obviously, when conducting surveys in community settings, the assessment tool should be easily managed by non-specialists and save time. Therefore, Jørgensen et al. substituted cognitive testing (Supermarket Fluency and Category Cued Memory Test) with questions regarding orientation, and developed a questionnaire version based on BASIC for community settings, the Brief Assessment of Impaired Cognition Questionnaire (BASIC-Q). The questionnaire included three components: self-report, orientation, and informant report, a total of 10 items; its sensitivity was 0.92 and its specificity was 0.97 to detect cognitive impairment, both of which were significantly higher than the sensitivity and specificity of the MMSE (28). In addition, its measurement time is short, which effectively reduces the survey burden on investigators and participants. Currently, there is no research to verify its use on the Chinese population. In only one study, the BASIC was translated into Chinese, and it was validated in stroke patients at a stroke treatment center (29). However, as we mentioned earlier, the Chinese version of BASIC is also not suitable for community settings.

In addition, to the best of our knowledge, no research has focused on the measurement invariance of the BASIC-Q. As a prerequisite for group comparison, measurement invariance refers to whether the meaning of measurement is equivalent between different groups (30). When comparing between groups, only the structure of the measurement was invariant between different groups, and statistical inference could be made (31). Considering that cognitive functions are easily affected by demographic factors such as sex, age, and education level, it is necessary to test the BASIC-Q for measurement invariance across sociodemographic factors.

In large-scale epidemiological surveys with more research content and larger sample sizes, short survey tools can reduce the workload of the survey, reduce the burden on the surveyed and the response bias, and are widely demanded by researchers. Based on the above considerations, in order to provide Chinese researchers with a shorter cognitive function assessment tool, this study aims to verify the Chinese version of the BASIC-Q (C-BASIC-Q) and explore its psychometric properties and measurement invariance.

## Materials and Methods

### Participants

From March to May 2021, a cross-sectional study of Chinese community-dwelling older adults was conducted in Jinan. A stratified cluster random sampling method was used to select the participants. First, using the 2020 annual per capita gross domestic product (GDP) level of the districts or counties of Jinan City, we divided the 12 districts and counties into three levels: high, medium, and low, with two districts or counties randomly selected from each level. Second, we randomly selected two townships or streets in the six selected districts or counties; therefore, a total of 12 streets or townships were selected. Third, we selected all older adults in two communities from the 12 randomly selected streets or towns to participate in a survey. Participants were included in the survey based on the following criteria: that they were 60 years or older, had lived in the area for more than 6 months, had no hearing or language impairment (self-reported), and voluntarily participated in the survey. Older persons who were clinically diagnosed with severe and terminal diseases or severe cognitive impairment, such as dementia (reported by family members), were excluded.

Before the survey, we communicated with community staff by telephone and determined the investigation time after obtaining their informed consent. We recruited participants by placing posters on publicity boards in the communities. To facilitate the inclusion of participants who were unable to read the questionnaire due to a low educational level, all questionnaires were completed by a uniformly trained investigator, using a face-to-face interview, instead of being filled out by the older adults themselves. A total of 2,201 participants who met the criteria were surveyed. After excluding invalid questionnaires with a wide range of missing content, 2,144 older adults (ranging in age from 60–99 years) participated in this study. The uniformly trained investigators completed the interview survey with the uniformly instructed language. The investigators were all medical undergraduates of grade 3 or above. It should be noted that in the factor structure analysis, we randomly divided the sample into two parts. Sample A (*n* = 1,072) consisted of 538 men and 534 women and was used for exploratory factor analysis. Sample B (*n* = 1,072) consisted of 537 men and 535 women and was used for confirmatory factor analysis. Moreover, 129 participants (67 men and 62 women, *M* = 72.9 years, *SD* = 7.1 years, ranging from 60–95 years) were selected to complete the retest of the C-BASIC-Q 2 weeks later. Unless otherwise specified, other reliability and validity indicators were based on the complete sample (*N* = 2,144).

All research procedures followed the principles of the Declaration of Helsinki. The study was approved by the Medical Ethics Committee of Xiangya School of Public Health, Central South University (identification code: XYGW-2020-101).

### Translation Procedure

We contacted Dr. Kasper Jørgensen by email and obtained his consent to translate the BASIC-Q into Chinese. First, two psychiatry graduate students were invited to independently translate the English version of the BASIC-Q into Chinese. Second, two professors with more than 10 years' experience in cognitive function research combined the existing Chinese versions of the BASIC to integrate the Chinese version of the questionnaire translated by two graduate students and produced a draft. Third, two English teachers with teaching experience in English-speaking countries and who were not familiar with the scale jointly translated the Chinese version of the scale into English and compared it with the original English version. They confirmed that the sentences and meanings of the translated English version were essentially the same as the original English version. Finally, the C-BASIC-Q was produced and used to evaluate cognitive functioning in the older Chinese adults in our sample.

### Measures

#### Brief Assessment of Impaired Cognition Questionnaire (BASIC-Q)

BASIC-Q was developed by Jørgensen et al. (28), which included three components: self-report (three items), orientation (four items), and informant report (3 items), a total of 10 items. Among them, self-report component uses three-category scoring method (0= *to a great extent*, 1= *to some extent*, 2= *no*). In the orientation component, two scores are possible (0 = wrong answer, 2 = correct answer). The informant report component also uses three-category scoring method (0= *much worse*, 1= *a bit worse*, 2= *unchanged*). The sum of all items gave the total score, ranging from 0 to 20 points. Higher scores indicated lower risks of cognitive impairment. Optimal cutoff score for case-finding of cognitive impairment was 16/17 in a previous study (28).

#### Mini-Mental State Examination (MMSE)

The MMSE was designed by Folstein et al. (32) to assess participants' cognitive function. The scale includes five dimensions: orientation, short-term memory, attention and calculation ability, recall ability, and language ability. Depending on the participants' answers, two scores are possible (0 = *wrong or unable to answer* or 1 = *true*). The sum of all items constituted the total score, ranging from 0 to 30 points. Higher scores indicated better cognitive functioning. With regard to the education level, the MMSE cut-off score for having MCI was <20 points for the illiterate group, <25 points for the primary school group, and <28 for the middle school and higher group (33). In this study, we used the Chinese version of the MMSE (34); Cronbach's alpha was 0.902.

#### Covariates

We used demographic characteristics, frequently used in previous studies of Chinese older adults (35), as covariates. The covariates were the following: age, sex, residence, education level, and marital status through a self-designed questionnaire survey. Age was categorized into three groups (60–69 years, 70–79 years, and 80 years and above). Residence was categorized as urban vs. rural. Educational level was categorized into three groups (illiteracy, primary school, and middle school and higher). Marital status was categorized as unmarried or married.

### Statistical Methods

SPSS version 25 (IBM SPSS Statistics, Armonk, NY, USA) was used to conduct descriptive statistical analysis, Student's *t*-tests, Pearson's r correlations, internal consistency tests and linear regression. Mplus version 8 was used to conduct the exploratory and confirmatory factor analyses. First, we ranked all participants according to their total C-BASIC-Q score from low to high, selecting the first 27% of participants as the low group, and the last 27% of participants as the high group. Item analysis was performed by comparing the score difference of each item in the high and low groups (used student's *t*-tests), and the correlation between each item score and the total score. The purpose of item analysis was to determine the homogeneity and discrimination of items to determine whether items need to be deleted. When the results of *t* test and correlation analysis were statistically significant, it indicated that all items had good homogeneity and differentiation.

Second, we used exploratory factor analysis (EFA) and confirmatory factor analysis (CFA) to evaluate the factor structure of the C-BASIC-Q. Specifically, we first used Mplus software to conduct EFA based robust maximum likelihood and explored three competition models: one factor, two factors, and three factors. Based on the fitting index, we selected an optimal model for the CFA. Given that the chi-square index is sensitive to sample size, the comparative fit index (CFI), Tucker–Lewis index (TLI), root-mean square error of approximation (RMSEA), and standardized root-mean square residual (SRMR) were used to evaluate model fit. The TLI and CFI are >0.900, and the RMSEA and SRMR are <0.080, indicating that the model fit well (36).

Convergent validity was evaluated by correlation with the MMSE score. Internal consistency and test-retest reliability were evaluated using Cronbach's alpha and retest correlations in a sub-sample (*n* = 129). In addition, in order to explore which MMSE and C-BASIC-Q scores were less affected by education level and age, we used linear regression models to analyze the impact of demographic factors on the MMSE and C-BASIC-Q scores. Finally, measurement invariance was evaluated using multi-group CFA. There are four levels of measurement invariance: configural, metric, scalar, and strict (37). Previous literature reviews suggested that scalar invariance was sufficient to evaluate the comparison between the mean values of latent factors, while strict invariance was more suitable for comparison between the mean values of observed factors (38, 39). Considering that the total score is generally used when comparing the cognitive function of participants with different demographic characteristics, we did not perform a strict invariance test. According to the fit criterion of the measurement invariance model (40), when the change in CFI (ΔCFI) and RMSEA (ΔRMSEA) is <0.010, compliance invariance is indicated.

## Results

### Descriptive Statistics

The descriptive statistics for the participants are presented in Table 1.

**Table 1:** Descriptive statistics for the participants.

**Variables**	**Total sample**
	**(*N* = 2,144)**
	***N*** **(%)/Mean ±SD**
**Age (years)**	72.01 ± 6.96
60–69	753 (35.1)
70–79	1,069 (49.9)
≥80	322 (15.0)
**Sex**	
Male	1,075 (50.1)
Female	1,069 (49.9)
**Residence**	
Urban area	829 (38.7)
Rural area	1,315 (61.3)
**Educational level**	
Illiteracy	509 (23.7)
Primary school	820 (38.2)
Middle school and above	815 (38.0)
**Marital status**	
Unmarried	498 (23.2)
Married	1,646 (76.8)
C-BASIC-Q	15.94 ± 3.43
MMSE	27.74 ± 3.89
Possible MCI (based on MMSE-score^*^)	
Yes	274 (12.8)
No	1,870 (87.2)

* *MMSE cutoff-scores for possible MCI: Illiterate group <20, primary school group < 25, middle school and higher group <28; SD, Standard deviation*.

### Item Analysis

The t-test results showed that the score differences of all items of the C-BASIC-Q in the high and low groups were statistically significant. Correlation analysis showed that the scores of all items were significantly and positively correlated with the total score. The data are shown in Table 2.

**Table 2:** Correlation analysis between each item of C-BASIC-Q and the total score of the scale and the results of the high and low group *t*-test (*N* = 2,144).

**Items**	***r***	***t***
1	0.664^***^	33.418^***^
2	0.749^***^	49.685^***^
3	0.742^***^	50.849^***^
4	0.649^***^	9.973^***^
5	0.602^***^	7.472^***^
6	0.663^***^	14.857^***^
7	0.655^***^	8.098^***^
8	0.703^***^	60.733^***^
9	0.712^***^	51.087^***^
10	0.625^***^	32.803^***^

*** *P < 0.001*.

### Structural Validity

The fit results of the three competing models obtained using EFA are shown in Table 3. Of the three, the fit index of the three-factor structure model was significantly better than that of both the two-factor and the single-factor structures. Therefore, we used the three-factor structure for the CFA. A preliminary analysis suggested that the initial CFA model of C-BASIC-Q has a poor fit index, so we set a residual correlation (item five with item seven) to correct the model. The results showed that the fit index of the corrected three-factor structure met the model-fit requirements. Above all, the C-BASIC-Q suggested a three-factor structure, namely self-report, orientation, and informant report. The loading of each item of the three-factor structure of the EFA and CFA is presented in Table 4.

**Table 3:** EFA and CFA fitting indexes of the C-BASIC-Q.

**Model**	**Factors**	**χ^2^**	***df***	**CFI**	**TLI**	**RMSEA**	**SRMR**
EFA	Single-factor	1838.198	35	0.655	0.556	0.219	0.143
EFA	Two-factor	509.436	26	0.907	0.840	0.132	0.058
EFA	Three-factor	69.282	18	0.990	0.975	0.052	0.017
CFA	Three-factor	186.956	31	0.970	0.956	0.069	0.040

**Table 4:** Loadings and commonality of each item on three factors in EFA and CFA.

**Items**	**Within sample A (*****N*** **= 1,072)**			**Within sample B (*****N*** **= 1,072)**		
	**Factor 1**	**Factor 2**	**Factor 3**	**Factor 1**	**Factor 2**	**Factor 3**
Item 1	**0.479**	−0.009	0.378	**0.653**		
Item 2	**0.571**	0.019	0.407	**0.790**		
Item 3	**0.593**	−0.008	0.421	**0.766**		
Item 4	0.338	**0.629**	−0.015		**0.831**	
Item 5	−0.008	**0.863**	−0.001		**0.764**	
Item 6	0.156	**0.496**	0.186		**0.687**	
Item 7	−0.012	**0.908**	0.062		**0.804**	
Item 8	−0.022	−0.029	**0.860**			**0.814**
Item 9	−0.002	0.030	**0.807**			**0.771**
Item 10	0.009	0.151	**0.543**			**0.609**

*The bold values indicate the corresponding items belonging to which factor*.

### Convergent Validity

The results of correlation analysis showed that the total C-BASIC-Q score was significantly positively associated with the total MMSE score (*r* = 0.590, *p* < 0.001).

### Internal Consistency and Test-Retest Reliability

Cronbach's alphas of the C-BASIC-Q and its three factors (self-report, orientation, and informant reports) were 0.862, 0.784, 0.840, and 0.782 in the total sample. The test-retest correlation coefficient was significant (*r* = 0.952, *p* < 0.001) in the retest sample (*n* = 129), indicating that the C-BASIC-Q has good internal consistency and test-retest reliability.

### Measurement Invariance

Table 5 shows the fit index of the measurement invariance model of the C-BASIC-Q's three-factor model across sex, age, residence, education level, and marital status. All model fit indices meet the fit standard. In addition, from the configural invariance model to the metric invariance model, and then to the scalar invariance model, the ΔCFI and ΔRMSEA were all <0.010. These results supported the identification of configural, metric, and scalar measurement invariance in the C-BASIC-Q across sex, age, residence, education level, and marital status.

**Table 5:** Measurement invariances across sex, age, residence, educational level, and marital status.

**Models**	**Measurement invariance**	**χ^2^**	**df**	**RMSEA**	**CFI**	**TLI**	**ΔRMSEA**	**ΔCFI**
Sex	Configural invariance	409.762	62	0.072	0.966	0.951		
	Metric invariance	430.024	69	0.070	0.965	0.954	0.002	0.001
	Scalar invariance	443.470	76	0.067	0.964	0.958	0.003	0.001
Age	Configural invariance	470.598	93	0.075	0.962	0.944		
	Metric invariance	495.972	107	0.071	0.960	0.950	0.004	0.002
	Scalar invariance	524.237	121	0.068	0.959	0.954	0.003	0.001
Residence	Configural invariance	422.550	62	0.074	0.965	0.949		
	Metric invariance	433.744	69	0.070	0.964	0.954	0.004	0.001
	Scalar invariance	441.485	76	0.067	0.964	0.958	0.003	<0.001
Educational level	Configural invariance	458.997	93	0.074	0.963	0.947		
	Metric invariance	529.441	107	0.074	0.957	0.946	<0.001	0.006
	Scalar invariance	565.399	121	0.072	0.955	0.950	0.002	0.002
Marital status	Configural invariance	388.539	62	0.070	0.969	0.954		
	Metric invariance	404.992	69	0.067	0.968	0.958	0.003	0.001
	Scalar invariance	423.871	76	0.065	0.966	0.960	0.002	0.002

### Factors Related to C-BASIC-Q and MMSE

The status of the C-BASIC-Q according to different participant characteristics is presented in Table 6. The results of linear regression analysis showed that age, residence, education level, and marital status all had an impact on the C-BASIC-Q and the MMSE scores (Table 7). By comparing the coefficients of determination, the impact of the four factors on the C-BASIC-Q was less than the impact on the MMSE (0.076 vs. 0.123).

**Table 6:** The status of C-BASIC-Q by different characteristics.

**Variables**	**Mean**	**SD**	***t/F***	***P*** **-value**
**Age (years)**			38.482	<0.001
60–69	16.52	3.00		
70–79	15.94	3.29		
≥80	14.55	4.33		
**Sex**			4.033	<0.001
Male	16.23	3.35		
Female	15.64	3.48		
**Residence**			5.374	<0.001
Urban area	16.42	3.12		
Rural area	15.63	3.58		
**Educational level**			41.279	<0.001
Illiteracy	14.88	4.10		
Primary school	15.92	3.25		
Middle school and above	16.61	2.95		
**Marital status**			6.821	<0.001
Unmarried	14.93	3.91		
Married	16.24	3.21		

*SD, Standard deviation*.

**Table 7:** Linear regression analysis for the contributions of sex, age, residence, education level, and marital status on C-BASIC-Q and MMSE.

**Factors**	**C-BASIC-Q**				**MMSE**			
	**B**	**SE of B**	**β**	***P*** **-value**	**B**	**SE of B**	**β**	***P*** **-value**
Sex	−0.207	0.151	−0.030	0.171	−0.269	0.167	−0.035	0.108
Age	−0.690	0.108	−0.137	<0.001	−0.461	0.119	−0.080	<0.001
Residence	−0.446	0.154	−0.063	0.004	−0.581	0.170	−0.073	0.001
Education level	0.583	0.102	0.131	<0.001	1.161	0.113	0.231	<0.001
Marital status	0.842	0.177	0.104	<0.001	1.296	0.195	0.141	<0.001
*R* ^2^	0.076				0.123			
*F* value	35.049^*^				60.033^*^			

*B, unstandardized regression coefficients; SE, standardized error; β, standardized regression coefficients; R^2^, coefficient of determination;*

* *P < 0.001*.

## Discussion

In this study, the BASIC-Q was translated into Chinese for the first time, and it was verified among community-dwelling older adults. This study provides scientific evidence for the application of the C-BASIC-Q in Chinese older adults and even other Chinese speaking people outside China. Our results showed that C-BASIC-Q has a three-factor structure, showing good structural validity. The C-BASIC-Q score was significantly correlated with MMSE, had good convergent validity, and its internal consistency and retest reliability were both good. In addition, its configural, metric, and scalar measurement invariance across sex, age, residence, education level, and marital status were supported. In general, the C-BASIC-Q has good reliability, validity, and measurement invariance, and can be used to evaluate the cognitive functioning of community-dwelling older adults in China.

The results of item analysis showed that each item was significantly positively associated with the C-BASIC-Q total score, and the difference in the scores of each item in the high and low groups was statistically significant, indicating that each item had good homogeneity and discrimination. Because factor structure analysis was not performed in the development of the original scale (28), we divided the total sample into two parts to explore the factor structure of the C-BASIC-Q, which is one of the novel contributions of this study. The results suggest that C-BASIC-Q has a three-factor structure and good structural validity. Interestingly, the items of the three factors of the C-BASIC-Q confirmed the three components of the original scale (i.e., self-report, orientation, and informant report). Therefore, the original three component names were adopted for the Chinese version. Moreover, correlation analysis showed that the C-BASIC-Q was significantly positively correlated with the MMSE; that is, the higher the C-BASIC-Q score, the better the cognitive functioning, showing good convergent validity. In addition, Cronbach's α coefficient of the C-BASIC-Q was 0.863, and the test-retest reliability at the two-week interval was 0.952, indicating that C-BASIC-Q has good internal consistency and stability, and the reliability was good.

Another important contribution of this study is the test of the measurement invariance of the C-BASIC-Q. Future studies can analyze the potential mean differences between different groups by determining the measurement invariance of the C-BASIC-Q. This study found that the C-BASIC-Q had configural, metric, and scalar measurement invariance across sex, age, residence, education level, and marital status. Specifically, the establishment of configural invariance means that the factor structure of the C-BASIC-Q between different groups was the same. Second, the results of metric invariance tests showed that the factor loadings between different groups were equivalent, that is, participants in different groups have the same understanding of each item. Finally, the results of scalar invariance suggested that the intercept of the C-BASIC-Q has cross-group equivalence, that is, the measurement properties of the C-BASIC-Q are the same across different groups. In summary, the evidence of the three invariance measurements indicated that the scores of the C-BASIC-Q are comparable regardless of sex, age, residence, education level, and marital status.

In this study, the differences in the C-BASIC-Q scores of participants of different age, sex, residence, education level, and marital status were statistically significant. Specifically, participants who were younger, male, living in urban areas, had higher education levels, and were married had higher cognitive functioning, which was consistent with the results of previous studies (33, 35). The results of linear regression analysis suggested that age, residence, education level, and marital status all had an impact on the C-BASIC-Q and the MMSE scores. However, it is worth noting that the variance explained by the above four factors on the C-BASIC-Q was lower than on the MMSE. In other words, compared with the MMSE, the C-BASIC-Q was less affected by age, residence, education, and marital status. This means the C-BASIC-Q is more suitable for epidemiological investigations, than the MMSE.

This study had several limitations. First, we did not have a gold standard for identifying MCI and dementia in this sample (that is, the clinician's diagnosis), which made it impossible to infer the cut-off value of the C-BASIC-Q for judging MCI and dementia. In the future, it will be necessary to combine the diagnosis of clinicians with the C-BASIC-Q, and determine the cut-off value of the C-BASIC-Q, which will lay the foundation for future MCI and dementia screening. Second, the sample for this study was drawn from only one city in China, which may not be nationally representative. In the future, scholars can expand the scope of sampling, increase the sample size, and further verify the C-BASIC-Q nationwide in China. Third, this study only verified the C-BASIC-Q in community settings, and it is necessary to conduct psychometric tests in the clinical environment or nursing home environment. Fourth, it has poor adaptability among elderly people living alone because the C-BASIC-Q includes an informant report. In situations where reliable informant reports cannot be obtained, a prorated BASIC-Q score may be used, but with a possible reduction in validity. Finally, since we only selected 129 participants to retest after 2 weeks, the sample size was small and the time-span was short, so a longitudinal invariance test was not performed.

Despite the above-mentioned limitations, this research is groundbreaking in the exploration of the C-BASIC-Q's factor structure and measurement invariance, and provides a reference for future validation of the BASIC-Q in other languages. In addition, we have provided a short, easy-to-use measurement tool for conducting surveys on the cognitive functioning of older adults in the Chinese community, and this is also a starting point for the use of the C-BASIC-Q in the measurement of cognitive functioning in China.

## Conclusions

The C-BASIC-Q is a valid tool for evaluating cognitive function in Chinese community-dwelling older adults with good reliability, validity, and measurement invariance.

## Data Availability Statement

The datasets can be made available to any interested person(s) contacting the corresponding author via email.

## Ethics Statement

The studies involving human participants were reviewed and approved by Medical Ethics Committee of Xiangya School of Public Health, Central South University. The patients/participants provided their written informed consent to participate in this study.

## Author Contributions

SL and GC designed the study, conducted surveys, analyzed data, and wrote the first manuscript. KJ provided suggestions on additional statistical analysis and manuscript writing and revised the original manuscript. ZC and ZL contributed to the scale translation process. HX revised the manuscript. All authors read and approved the final manuscript.

## Conflict of Interest

The authors declare that the research was conducted in the absence of any commercial or financial relationships that could be construed as a potential conflict of interest.

## Publisher's Note

All claims expressed in this article are solely those of the authors and do not necessarily represent those of their affiliated organizations, or those of the publisher, the editors and the reviewers. Any product that may be evaluated in this article, or claim that may be made by its manufacturer, is not guaranteed or endorsed by the publisher.

## Abbreviations

C-BASIC-Q: Chinese version of the Brief Assessment of Impaired Cognition Questionnaire
MMSE: Mini-Mental State Examination
MCI: Mild cognitive impairment
EFA: Exploratory Factor Analysis
CFA: Confirmatory Factor Analysis
CFI: Comparative Fit Index
TLI: Tucker–Lewis Index
RMSEA: Root-Mean Square Error of Approximation
SRMR: Standardized Root-Mean Square Residual.
